# 111 The Association of Burn Center Profit Status and Trauma Designation with Racial/Ethnic Diversity and Transfers

**DOI:** 10.1093/jbcr/irae036.110

**Published:** 2024-04-17

**Authors:** Samantha Steeman, Devi Lakhlani, Eloise Stanton, Clifford C Sheckter

**Affiliations:** Stanford University, Stanford, California; Keck School of Medicine of USC, Los Angeles, CA; Stanford/Santa Clara Valley Medical Center, San Jose, CA; Stanford University, Stanford, California; Keck School of Medicine of USC, Los Angeles, CA; Stanford/Santa Clara Valley Medical Center, San Jose, CA; Stanford University, Stanford, California; Keck School of Medicine of USC, Los Angeles, CA; Stanford/Santa Clara Valley Medical Center, San Jose, CA; Stanford University, Stanford, California; Keck School of Medicine of USC, Los Angeles, CA; Stanford/Santa Clara Valley Medical Center, San Jose, CA

## Abstract

**Introduction:**

Health equity has become a prominent focus in conversations on improving healthcare delivery. Concerns have arisen that for-profit burn centers without trauma status may engage in patient selection practices that favor commercially insured patients, potentially resulting in underrepresentation of minority patients. This study investigates the relationship between profit status and trauma center designation in burn centers in relation to diversity of patients treated.

**Methods:**

Using the California Department of Health Care Access and Information (HCAI), 2009-2019, all inpatient encounters at verified and non-verified burn centers were identified using ICD-9/10 and diagnosis related group codes. Primary outcomes of interest included proportion of burn transfers accepted with Medi-Cal or uninsured and the proportion of minority patients treated. Key covariates included burn center safety net status, profit status, American Burn Association (ABA) verification status, and American College Surgeons (ACS) Level 1 trauma center designation. Adjusted covariates were burn size, gender, age, and payer type. Linear regression modeling was performed and analyses were conducted at the level of individual burn centers, annually. Bonferroni adjustments were applied for multiple testing.

**Results:**

There were 12 burn centers over 11 years. The median proportion of Black and Hispanic patients treated per burn center per year was 44.7%, (IQR 37.4%, 53.2%). On multivariable analysis, for profit burn centers were negatively associated with proportion of Black and Hispanic patients treated (coef -9.2%, p=0.008), and burn centers with trauma center designation were positively associated with higher proportion (coef 8.1%, p=0.011). Safety-net status and verification did not have a significant relationship, though safety-net burn centers care for significantly more MediCal (Medicaid) patients (coef 9.9%, p=0.001). There was not a single ED to burn center transfer for an uninsured patient at for-profit burn center, compared to 9 uninsured transfers to non-profit burn centers. There were only 2 ED to burn center transfers for MediCal (Medicaid) patients at for-profit centers compared to 239 for non-profit burn centers.

**Conclusions:**

For-profit burn centers care for disproportionately few Black and Hispanic patients, while burn centers with trauma center designation care for more. For-profit burn centers have disproportionately fewer transfers with Medi-Cal (Medicaid) payer or uninsured compared to other burn centers.

**Applicability of Research to Practice:**

In the pursuit of delivering high equity burn care across the US, this study identifies disparities related to profit status and trauma center designation in burn centers, underscoring the need for targeted, equity-based interventions and policy development.

